# The Illusion of Distribution-Free Small-Sample Classification in Genomics

**DOI:** 10.2174/138920211796429763

**Published:** 2011-08

**Authors:** Edward R Dougherty, Amin Zollanvari, Ulisses M Braga-Neto

**Affiliations:** 1 Department of Electrical and Computer Engineering, Texas A&M University; 2 Computational Biology Division, Translational Genomics Research Institute; 3 Department of Bioinformatics and Computational Biology, University of Texas M. D. Anderson Cancer Center; 4 Children’s Hospital Informatics Program at Harvard-MIT Division of Health Sciences and Technologies, Brigham and Women’s Hospital and Harvard Medical School, USA

**Keywords:** Classification, epistemology, error estimation, genomics, validation.

## Abstract

Classification has emerged as a major area of investigation in bioinformatics owing to the desire to discriminate phenotypes, in particular, disease conditions, using high-throughput genomic data. While many classification rules have been posed, there is a paucity of error estimation rules and an even greater paucity of theory concerning error estimation accuracy. This is problematic because the worth of a classifier depends mainly on its error rate. It is common place in bio-informatics papers to have a classification rule applied to a small labeled data set and the error of the resulting classifier be estimated on the same data set, most often *via* cross-validation, without any assumptions being made on the underlying feature-label distribution. Concomitant with a lack of distributional assumptions is the absence of any statement regarding the accuracy of the error estimate. Without such a measure of accuracy, the most common one being the root-mean-square (RMS), the error estimate is essentially meaningless and the worth of the entire paper is questionable. The concomitance of an absence of distributional assumptions and of a measure of error estimation accuracy is assured in small-sample settings because even when distribution-free bounds exist (and that is rare), the sample sizes required under the bounds are so large as to make them useless for small samples. Thus, distributional bounds are necessary and the distributional assumptions need to be stated. Owing to the epistemological dependence of classifiers on the accuracy of their estimated errors, scientifically meaningful distribution-free classification in high-throughput, small-sample biology is an illusion.

## INTRODUCTION

The advent of high-throughput genomic data has brought a host of proposed classification rules to discriminate types of pathology, stages of a disease, duration of survivability, and other phenotypic discriminations. Using gene expression as archetypical, these generally follow a common methodology: (1) identify each expression profile (feature vector) within the data set with a class, meaning that a label is associated with each profile, (2) use a classification rule, including feature selection, to design a classifier, and (3) use an error estimation rule to estimate the error of the designed classifier. A critical issue, and one not explicitly stated, is that the entire procedure is done without any assumptions on the feature-label distribution (population). This issue is critical because the performances of both the classification and error estimation rules depend heavily on the population, specifically, the class-conditional distributions governing the profiles and the labels. It may be argued that one can apply any classification rule, without concern for the feature-label distribution, because ultimately it is the error of the designed classifier that matters and, if one uses an inappropriate classification rule, then the price will be paid in poor performance. While ignoring the properties of a classification rule may not be the most prudent way to go about designing classifiers, there is no epistemological difficulty in doing so. On the other hand, since the worth of a classifier rests with its error, error estimation performance is crucial.

When an error estimate is reported, it implicitly carries with it the properties of the error estimator; otherwise, the estimate carries no knowledge. If no distribution assumptions are made, then very little, or perhaps nothing, can be said about the precision of the estimate. In the rare instances in which performance bounds are known in the absence of any assumptions on the feature-label distribution, those bounds are so loose as to be virtually worthless for small samples. Consequently, if the authors are claiming that the error estimate carries any knowledge, then they are implicitly making distributional assumptions. The implicit nature of the assumptions invalidates the entire enterprise. It is precisely the explicitness of assumptions that renders the conclusion meaningful.

### Classifier Models

For two-class classification, the population is characterized by a feature-label distribution *F* for a random pair (**X**, *Y*), where **X** is the vector of features (gene expression vector in the case of microarrays) and *Y* is the binary label, 0 or 1, of the class containing **X**. A classifier is a function ψ(**X**) which assigns a binary label to each feature vector. The error, ε[ψ], of a classifier ψ is the probability that ψ produces an erroneous label. A classifier with minimum error among all classifiers is known as a *Bayes classifier* for the feature-label distribution and this minimum error is known as the *Bayes error*. From an epistemological perspective, the error is the key issue since it quantifies the predictive capacity of the classifier and scientific validity is characterized by prediction [1]. One can apply the same classifier to any number of feature-label distributions and the error for a particular distribution characterizes classifier prediction on that distribution.

Abstractly, any pair *M* = (ψ, ε_ψ_) composed of a function $\psi :{ℜ}^{d}\to \left\{0,1\right\}$ and a real number $\varepsilon_{\psi }\in \left[0,1\right]$ constitutes a *classifier model*, with ε_ψ_ not specifying an actual error probability corresponding to ψ. *M* becomes a scientific model when it is applied to a feature-label distribution. At this point model validity comes into question. Irrespective of where ψ comes from, the model is valid for the feature-label distribution *F* to the extent that ε_ψ_ approximates the classifier error, ε[ψ], on *F*. The degree of approximation must be measured by some distance-type function, $\delta \left(\varepsilon_{\psi },\varepsilon \left[\psi \right]\right)$, between ε_ψ_ and ε[ψ], such as the absolute difference |ε_*f*_[ψ] – ε_ψ_|.

In practice the feature-label distribution is unknown and a *classification rule* Ψ_*n*_ is used to design a classifier ψ_*n*_ from a random sample *S_n_* = {(**X**_1_, *Y*_1_), (**X**_2_, *Y*_2_),…, (**X**_*n*_, *Y_n_*)} of pairs drawn from the feature-label distribution. Note that a classification rule is really a sequence of classification rules depending on the sample size *n*. If feature selection is involved, then it is part of the classification rule. A designed classifier produces a classifier model, namely, (ψ_*n*_, ε[ψ_*n*_]). Since the true classifier error ε[ψ_*n*_] depends on the feature-label distribution, which we do no know, ε[ψ_*n*_] is unknown. In practice, the true error is estimated by an *estimation rule*, Ξ_*n*_. Thus, the random sample *S_n_* yields a classifier ψ_*n*_ = Ψ_*n*_(*S_n_*) and an error estimate $\overset{\wedge }{\varepsilon }\left[\psi_{n}\right]=\Xi_{n}\left(S_{n}\right)$, which together constitute a classifier model $\left(\psi_{n},\overset{\wedge }{\varepsilon }\left[\psi_{n}\right]\right)$. In sum, practical classifier design involves a *rule model* (Ψ_*n*_, Ξ_*n*_) used to determine a sample-dependent classifier model $\left(\psi_{n},\overset{\wedge }{\varepsilon }\left[\psi_{n}\right]\right)$. Since the classifier depends on a random sample, both (ψ_*n*_, ε[ψ_*n*_]) and $\left(\psi_{n},\overset{\wedge }{\varepsilon }\left[\psi_{n}\right]\right)$ are random.

### Validity

Given a specific sample, ε[ψ_*n*_] and $\overset{\wedge }{\varepsilon }\left[\psi_{n}\right]$ are fixed values but we do not know ε[ψ_*n*_]; however, given a feature-label distribution, we can compute an expected distance between the estimated and true errors. Thus, model validity is characterized in terms of the performance of the rule model, that is, the precision of the error estimator $\overset{\wedge }{\varepsilon }\left[\psi_{n}\right]=\Xi_{n}\left(S_{n}\right)$ as an estimator of ε[ψ_*n*_]. That is, model validity is defined *via *the properties of the error estimation rule relative to the classification rule and the feature-label distribution. For notational ease we denote ε[ψ_*n*_] and $\overset{\wedge }{\varepsilon }\left[\psi_{n}\right]$ by ε and $\overset{\wedge }{\varepsilon }$, respectively. An obvious choice for measuring model validity is the expected absolute difference, namely, $E\left[\left|\overset{\wedge }{\varepsilon }-\varepsilon \right|\right]$; however, it is more common to use the *root-mean-square* (*RMS*) error, defined by

(1) $${\mathrm{RMS}}_{n}\left(\overset{\wedge }{\varepsilon }\right)=\sqrt{E\left[{\left|\overset{\wedge }{\varepsilon }-\varepsilon \right|}^{2}\right]}$$

The RMS can be decomposed into the bias, *Bias*$\left[\overset{\wedge }{\varepsilon }\right]=E\left[\overset{\wedge }{\varepsilon }-\varepsilon \right]$, of the error estimator relative to the true error, and the deviation variance, ${\mathrm{Var}}_{\mathrm{dev}}\left[\overset{\wedge }{\varepsilon }\right]=\mathrm{Var}\left[\overset{\wedge }{\varepsilon }-\varepsilon \right]$, according to

(2) $${\mathrm{RMS}}_{n}\left(\overset{\wedge }{\varepsilon }\right)=\sqrt{{\mathrm{Var}}_{\mathrm{dev}}\left[\overset{\wedge }{\varepsilon }\right]+\mathrm{Bias}{\left[\overset{\wedge }{\varepsilon }\right]}^{2}}$$

Since $E\left[\left|\overset{\wedge }{\varepsilon }-\varepsilon \right|\right]\leq {\mathrm{RMS}}_{n}\left(\overset{\wedge }{\varepsilon }\right)$, a small RMS guarantees a small expected absolute difference. If we use the RMS to characterize model validity, then the model with smaller RMS is more valid. Our goal is to have the RMS as small as possible.

Rather than consider the expectation of the squared absolute difference, one can require that the absolute difference is not too large with high probability. Letting the probability 0.95 (or some other value) represent strong confidence, we can measure validity by the value *r* > 0 that results in $P\left(\left|\overset{\wedge }{\varepsilon }-\varepsilon \right|>r\right)=0.05$. Whereas computation of the RMS requires only the first and second moments of the true and estimated errors, computation of this tail probability involves the joint distribution of the true and estimated errors. In this paper we confine ourselves to RMS but the epistemological concepts are immediately extendable to validity measured by the tail probability.

### Epistemology

Epistemologically, when a classifier is designed and an error estimate computed, model validity, and, hence, the degree to which the model has meaning, rests with the properties of the error estimator, in particular, the RMS or some other specified measure of validity [1]. Absent some quantitative measure of validity, a classifier model is epistemologically vacuous, that is, absent of meaning. In and of itself, an estimation rule is nothing more than a computation. Any number of computations can be proposed and, unless these are judged by some criterion, all are equally vacuous The criterion is a choice among researchers, there may be many criteria, and one classifier model may be more valid than another relative to one criterion and less valid relative to another. But a criterion must be posited for a classifier model to have any scientific meaning.

Suppose a sample is collected, a classification rule Ψ_*n*_ applied, and the classifier error estimated by an error-estimation rule Ξ_*n*_ to arrive at the classifier model $\left(\psi_{n},\overset{\wedge }{\varepsilon }\left[\psi_{n}\right]\right)$. If no assumptions are posited regarding the feature-label distribution, then it must be assumed that no such assumptions are being made and the entire procedure is completely distribution-free with respect to the feature-label distribution. There are three possibilities. First, if no validity criterion is specified, then the classifier model is *ipso facto* epistemologically meaningless. Simply put, there is no way to evaluate the classifier model. Second, suppose a validity criterion is specified, say RMS, and no distribution-free results are known about the RMS for Ψ_*n*_ and Ξ_*n*_. Again, the model is meaningless because nothing can be said about the performance of the error-estimation rule. Third, again assuming RMS as the measure of validity, suppose there exist distribution-free bounds concerning Ψ_*n*_ and Ξ_*n*_. Then these bounds can be used to quantify the performance of the error estimator and thereby quantify model validity.

Regarding the latter case, consider the leave-one-out error estimator, ${\overset{\wedge }{\varepsilon }}^{\mathrm{loo}}$, and the *k*-nearest-neighbor classification rule with random tie-breaking. There exists a distribution-free bound:

(3) $${\mathrm{RMS}}_{n}\left({\overset{\wedge }{\varepsilon }}^{\mathrm{loo}}\right)\leq \sqrt{\frac{1+24\sqrt{k/2\pi }}{n}}$$

[2]. If *k* = 3 and the sample size is *n* = 100, then the bound is approximately 0.353, so that there is very little model validity and knowledge of the true error is highly uncertain.

For leave-one-out error estimation, the histogram rule, and multinomial discrimination with *b* cells, there exists the following distribution-free bound:

(4) $${\mathrm{RMS}}_{n}\left({\overset{\wedge }{\varepsilon }}^{\mathrm{loo}}\right)\leq \sqrt{\frac{{1+6e}^{-1}}{n}+\frac{6}{\sqrt{\pi \left(n-1\right)}}}$$

[3]. If the sample size is *n* = 100, then the bound is approximately 0.601, so that there is very little model validity and knowledge of the true error is essentially nil. With such an RMS, even a very small estimate is of no value. If *n* = 10,000, then the RMS is approximately 0.184, which is still poor. Thus, distribution-free bounds such as those in Eqs. 3 and 4 have virtually no practical use.

Even if a feature-label distribution is assumed, estimation can still be very bad. Consider an arbitrary feature-label distribution and nearest-neighbor classification. For the resubstitution error estimator, ${\overset{\wedge }{\varepsilon }}^{\mathrm{res}}=0$, irrespective of the data. If ε_*bay*_ denotes the Bayes error, then ε ≥ ε_*bay*_ and

(5) $${\mathrm{RMS}}_{n}\left({\overset{\wedge }{\varepsilon }}^{\mathrm{res}}\right)=\sqrt{E\left[{\left|{\overset{\wedge }{\varepsilon }}^{\mathrm{res}}-\varepsilon \right|}^{2}\right]}=\sqrt{E\left[\varepsilon^{2}\right]}\geq \sqrt{E\left[\varepsilon_{\mathrm{bay}}^{2}\right]}=\varepsilon_{\mathrm{bay}}$$

While this situation is pathological, it reveals the importance of the Bayes error relative to RMS. If the Bayes error is 0, then it simply says that the RMS exceeds 0, so that it is possible the RMS is small and the resubstitution error is accurate. At the other extreme, if the Bayes error is 0.5, then the RMS exceeds 0.5. In general, the relationship between the RMS and the Bayes error is important for determining error estimation performance, not just in the case of resubstitution.

To examine the relationship between the RMS and Bayes error, we consider a feature-label distribution having two equally probable Gaussian class-conditional densities sharing a known covariance matrix and the linear discriminant analysis (LDA) classification rule. For this model the Bayes error is a one-to-one decreasing function of the distance, *m*, between the means. Moreover, for this model we possess analytic representations of the joint distributions of the true error with both the resubstitution and leave-one-out error estimators, exact in the univariate case and approximate in the multivariate case [4]. Whereas one could utilize these approximate representations to find approximate moments *via *integration, more accurate approximations, including the second-order mixed moment and the RMS, can be achieved for this Gaussian model *via *asymptotically exact analytic expressions using a double asymptotic approach, where both sample size and dimensionality approach infinity at a fixed rate between the two [5]. Finite-sample approximations from the double asymptotic method have long been known to show good accuracy [6, 7]. Figs. (**1** and **2**), computed based on the results in [5], show the RMS to be a one-to-one increasing function of the Bayes error for resubstitution and leave-one-out, respectively, for dimensions *p* = 5, 10, 25 and sample sizes *n *= 20, 40, 60, the RMS and Bayes errors being on the *y* and *x* axes, respectively. This monotonic behavior for the RMS as a function of the Bayes error is not uncommon (but not always the case).

Assuming a parameterized model in which the RMS is an increasing function of the Bayes error, we can pose the following question: Given sample size *n* and λ > 0, what is the maximum value, *maxBayes*(λ), of the Bayes error such that ${\mathrm{RMS}}_{n}\left(\overset{\wedge }{\varepsilon }\right)\leq \lambda ?$ If RMS is the measure of validity and λ represents the largest acceptable RMS for the classifier model to be considered meaningful, then the epistemological requirement is characterized by *maxBayes*(λ).Given the relationship between model parameters and the Bayes error, the inequality ε_*bay*_ ≤ *maxBayes*(λ) can be solved in terms of the parameters to arrive at a necessary modeling assumption.

In the preceding Gaussian example, since ε_*bay*_ is a decreasing function of *m*, we obtain an inequality of the form *m* ≥ *m*(λ). Figs. (**3** and **4**) show the *maxBayes*(λ) curves corresponding to the RMS curves in Figs. (**1** and **2**), respectively. These curves show that, even if one assumes Gaussian class-conditional densities and a known common covariance matrix, further assumptions must be made on the Bayes error, or, equivalently, on model parameters, to insure that the RMS is sufficiently small to make the classifier model meaningful. Absent a Gaussian or some other assumption of a distributional family, one could not even proceed to obtain a Bayes-error requirement.

We now consider the discrete histogram classification rule for multinomial discrimination with *b* bins under the assumption that the class-conditional probabilities are determined by a Zipf model with parameter α [8]. As α → 0, the distributions tend to uniformity, which represents maximum discriminatory difficulty. As α → ∞, the distributions become concentrated in single (distinct) bins, corresponding to maximum discrimination between the classes. The Bayes error is a decreasing function of α. We assume α is unknown; otherwise, we would know the feature-label distribution. The joint distributions of the true error with the leave-one-out and resubstitution estimators are known [9, 10] and closed-form expressions for the second moments are given in [11]. The RMS can be computed exactly based upon the formulas in the latter. Figs. (**5** and **6**), based on these, show the RMS for leave-one-out and resubstitution, respectively, as a function of the Bayes error for *b* = 4, 8, 16, and sample sizes *n *= 20, 40, 60. The RMS is greater for leave-one-out for *b* = 4, the RMS is greater for resubstitution for *b* = 16, and there is little RMS difference for *b* = 8. Figs. (**7** and **8**) show the *maxBayes*(λ) curves corresponding to Figs. (**5** and **6**), respectively. Assuming a Zipf model gives a one-to-one correspondence between α and the Bayes error, so that the inequality ε_*bay*_ ≤ *maxBayes*(λ) is equivalent to an inequality of the form α ≥ α(λ). We could skip the Zipf assumption but then the inequality ε_*bay*_ ≤ *maxBayes*(λ) would be equivalent to a region in the (*b *- 1)-dimensional space of the bin probabilities *p*_1_, *p*_2_,…, *p_b-1_*.

To illustrate the advantage of knowing the RMS based on distributional assumptions, consider the following RMS bound for the discrete histogram rule for resubstitution, where *b* is the number of cells and *n* the sample size:

(6) $${\mathrm{RMS}}_{n}\left({\overset{\wedge }{\varepsilon }}^{\mathrm{res}}\right)\leq \sqrt{\frac{6b}{n}}$$

[3]. Based on this bound, if *b* = 4, then the sample size must exceed 1667 to insure ${\mathrm{RMS}}_{n}\left({\overset{\wedge }{\varepsilon }}^{\mathrm{res}}\right)\leq 0.12$. If, on the other hand, we assume a Zipf discrete model and use the RMS resubstitution results in [11], then we find that a sample size of only 40 insures ${\mathrm{RMS}}_{n}\left({\overset{\wedge }{\varepsilon }}^{\mathrm{res}}\right)\leq 0.12$.

Because we have the Bayes errors and closed-form expressions for the RMS in the preceding examples, everything is done analytically and characterized relative to the Bayes error, which is a universal measure of classification difficulty. If the Bayes error is unknown, then the analysis can be performed using distribution parameters. In addition, we have been able to impose distributional assumptions so that there is a single parameter, say δ, such that ε_*bay*_ ≤ *maxBayes*(λ) if and only if δ ≥ δ(λ), or δ ≤ δ(λ). This condition simplifies matters, but is not necessary.

### Contra Intuition 

**Fig. (3):**
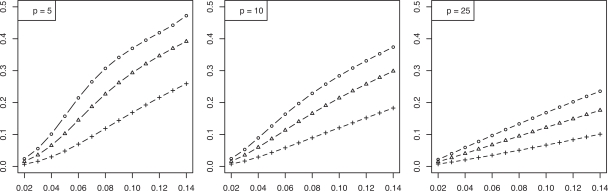
Maximum Bayes error *versus* RMS = λ for resubstitution in a Gaussian model: (+) is *n* = 20; (Δ) is *n* = 40; (o) is *n* = 60.

Absent knowledge of its properties, an error estimator is a meaningless computation. From a scientific perspective, the situation is no better if one justifies application of an error estimator on intuitive nonmathematical, or mathematically spurious, grounds. As an illustration, consider the argument that leave-one-out is unbiased. This argument is spurious because it omits the fact that bias is only one factor in error estimation performance – in particular, only one term in Eq. 2 for the RMS. There is also the deviation variance in Eq. 2. Not only does the unbiasedness of leave-one-out not guarantee good performance, but it does not even guarantee better performance than resubstitution (Fig. **3**). Arguments such as the approximate unbiasedness of leave-one-out demonstrate a disregard for sound epistemology. To emphasize this point, we will first consider some Monte-Carlo results from the 1970s and some error bounds, and then we will turn to more contemporary analytic results characterizing exact performance.

In a classic 1978 paper, Ned Glick considers LDA classification for one-dimensional Gaussian class-conditional distributions possessing unit variance, with means μ_0_ and μ_1_, and a sample size of *n* = 20 with an equal number of sample points from each distribution [12]. Fig. (**9**) is based on Glick’s paper; however, we have increased the Monte Carlo repetitions from 400 to 20,000 for increased accuracy. In both parts, the *x*-axis is labeled with *m* = |μ_0_ – μ_1_|, which is the Mahalanobis distance in this setting, with the parentheses containing the corresponding Bayes error. εbaym,EεLDAm,Eε∧resm, and Eε∧loom denote the Bayes error, the expected true error of the LDA classifier, the expected resubstitution error of the LDA classifier, and the expected leave-one-out error of the LDA classifier, respectively. Three curves are plotted in Fig. (**9a**): (1) *E*[ε_*LDA*_(*m*)] – ε_*bay*_(*m*) (solid), (2) $E\left[{\overset{\wedge }{\varepsilon }}^{\mathrm{res}}\left(m\right)\right]-\varepsilon_{\mathrm{bay}}\left(m\right)$ (dots), and (3) $E\left[{\overset{\wedge }{\varepsilon }}^{\mathrm{loo}}\left(m\right)\right]-\varepsilon_{\mathrm{bay}}\left(m\right)$ (dashes). Since the designed classifier cannot be better than the Bayes classifier,

(7) $$E\left[\varepsilon_{\mathrm{LDA}}\left(m\right)\right]-\varepsilon_{\mathrm{bay}}\left(m\right)>0.$$

Resubstitution is sufficiently optimistically biased as an estimator of ε_*LDA*_ that

(8) $$E\left[{\overset{\wedge }{\varepsilon }}^{\mathrm{res}}\left(m\right)\right]-\varepsilon_{\mathrm{bay}}\left(m\right)<0.$$

Leave-one-out is slightly pessimistically biased, so that

(9) $$E\left[{\overset{\wedge }{\varepsilon }}^{\mathrm{loo}}\left(m\right)\right]-\varepsilon_{\mathrm{bayt}}\left(m\right)\approx E\left[\varepsilon_{\mathrm{LDA}}\left(m\right)\right]-\varepsilon_{\mathrm{bay}}\left(m\right).$$

The salient point of Glick’s paper appears in Fig. (**9b**), which plots the standard deviations corresponding to εLDAm,ε∧resm, and ε∧loom using the same line coding. When the optimal error is small, the standard deviations of the leave-one-out error and the resubstitution error are close, but when the error is large, the leave-one-out error has a much greater standard deviation. Glick was sufficiently concerned that, with regard to the leave-one-out estimator, he wrote, “I shall try to convince you that one should not use this modification of the counting estimator (for the usual linear discriminant)” – not even for LDA in the Gaussian model. Glick’s concerns have been confirmed and extended beyond the Gaussian model in studies involving Monte Carlo simulations [13, 14] and in analytic results [4, 10], where it has been shown that for small samples the leave-one-out error estimator can be negatively correlated with the true error.

Let us close this section by illustrating how different error estimator comparison can be for small and large samples. In Eq. 4, (*n *– 1)^–1/4^ is the dominant term, whereas *n*^–1/2^ is dominant in Eq. 6. Thus, relative to the loose bounds in these equations, leave-one-out may have larger asymptotic RMS than resubstitution as *n* → ∞ for the discrete histogram rule. The curves in Fig. (**10**), based on the results in [11], show the RMS as a function of sample size for the Zipf model, with Bayes error 0.2. For *b* = 4, ${\mathrm{RMS}}_{n}\left({\overset{\wedge }{\varepsilon }}^{\mathrm{res}}\right)<{\mathrm{RMS}}_{n}\left({\overset{\wedge }{\varepsilon }}^{\mathrm{loo}}\right).$ For *b* = 8, ${\mathrm{RMS}}_{n}\left({\overset{\wedge }{\varepsilon }}^{\mathrm{res}}\right)>{\mathrm{RMS}}_{n}\left({\overset{\wedge }{\varepsilon }}^{\mathrm{loo}}\right)$ for *n* < 35 but ${\mathrm{RMS}}_{n}\left({\overset{\wedge }{\varepsilon }}^{\mathrm{res}}\right)<{\mathrm{RMS}}_{n}\left({\overset{\wedge }{\varepsilon }}^{\mathrm{loo}}\right)$ for *n* ≥ 35, which is in accord with the relations contained in Eqs. 4 and 6. For *b* = 16, ${\mathrm{RMS}}_{n}\left({\overset{\wedge }{\varepsilon }}^{\mathrm{res}}\right)>{\mathrm{RMS}}_{n}\left({\overset{\wedge }{\varepsilon }}^{\mathrm{loo}}\right)$ for the sample sizes shown, but the inequality will eventually flip. We observe that, for low complexity, resubstitution can outperform leave-one-out cross-validation for small samples. As complexity increases, leave-one-out tends to outperform resubstitution; however, asymptotically, as *n* → ∞, resubstitution will again outperform leave-one-out, a point made in [3]. Simple, supposedly intuitive, arguments are not going to obtain these results.

## CONCLUSION

Very rarely is there analytic knowledge of the joint distribution of the true and estimated errors, or the RMS, two instances being the Gaussian model with known common covariance matrix using linear discriminant analysis [4] and multinomial discrimination [9, 10]. While there have been some attempts to estimate the variance of an error estimator from the training data, these are generally ad hoc and have been demonstrated to be very inaccurate, and therefore of negligible value for quantifying error estimation accuracy [15]. Moreover, if one is to apply an RMS bound, this requires a distributional assumption, which in turn means that if one wishes to claim the benefit of a classification rule for a specific biological application, then either the application must be sufficiently understood so that the relevant variables can be assumed to obey, at least approximately, a known probabilistic law or some statistical test must be applied to provide reasonable assurance that the variables do not deviate significantly from the distributional assumptions under which the RMS bound is being computed.

What happens when one is confronted with a small sample and the features are not Gaussian or multinomial, or if one wishes to use error estimators for which nothing is known about the RMS? In the absence of analytic results, one could use Monte-Carlo techniques based on distributional assumptions to obtain bounds on the RMS. This approach would be heavily computational and would provide only a sampling of RMS values; nonetheless, it could provide useful information on the accuracy of error estimation if sufficient computational power were employed. Ultimately, of course, the problem is a lack of attention to small-sample theory. Prior to 1980, there was some interest in the accuracy of error estimation, mainly with regard to the first or second moments of resubstitution (see [4] for a compendium). While these revealed the optimistic bias of resubstitution in the models considered, they did not address the joint second moments between the true and estimated errors, which are needed for a deeper understanding of error estimation accuracy. Making matters worse, between 1980 and 2005 there was hardly any theoretical interest in error estimation accuracy. This lack of interest is surprising in that various enhancements of cross-validation, including bootstrap, were proposed, but apparently with little concern for their small-sample performance, which is especially surprising given that with large samples the data can be split into training and testing data, thereby precluding the need for error estimation on the training data.

Interestingly, the requirement of RMS bounds based on distributional assumptions follows from a recent statement made in an editorial in *Bioinformatics* written by several associate editors of the journal, when they write: “While simulation may still be worthwhile, and a useful tool for exploring robustness and parameter space of a new method, it is insufficient evidence for superiority of a new method without substantial support from significant improvement in results from analysis of real data” [16]. Significant improvement can only be demonstrated if there are bounds quantifying error estimation accuracy. This is an epistemological requirement and it lies at the heart of the classification-related epistemological problems in bioinformatics articulated in a number of papers [1, 17-24].

Small-sample classification is no place to rely on intuition, analogy, distribution-free asymptotic theory, or non-rigorous quasi-mathematical “propositions.” Heuristic or incomplete mathematical arguments regarding error estimation should be shunned and any claimed results should be evaluated on the basis of verified properties of error estimators. One should be particularly wary of distribution-free classifier models since it is extremely unlikely that the purported results possess any solid foundation and there is a good possibility that they are epistemologically meaningless or, at least, any meaning they do possess is unknown to even the claimants. In the case of leave-one-out, and other cross-validation techniques, it is perplexing that, given Glick’s stark warning, and recent reconfirmations, it has continued to be used up until the present day in small-sample settings in the absence of distributional assumptions.

While omitting distributional assumptions might lead one to believe that the results are more far reaching; in fact, this is typically an illusion because in small-sample settings the absence of distributional assumptions usually renders the entire study vacuous. Simply put, scientifically sound model-free classification is impossible in small-sample settings. Should one doubt this, consider the comment by R. A. Fisher in 1925 on the limitations of large-sample methods:

“Little experience is sufficient to show that the traditional machinery of statistical processes is wholly unsuited to the needs of practical research. Not only does it take a cannon to shoot a sparrow, but it misses the sparrow! The elaborate mechanism built on the theory of infinitely large samples is not accurate enough for simple laboratory data. Only by systematically tackling small sample problems on their merits does it seem possible to apply accurate tests to practical data [25]”.

## Figures and Tables

**Fig. (1):**
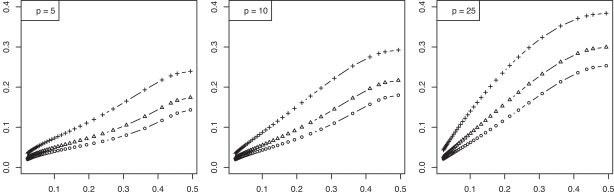
RMS *versus* Bayes error for resubstitution in a Gaussian model: (+) is *n* = 20; (Δ) is *n* = 40; (o) is *n* = 60.

**Fig. (2):**
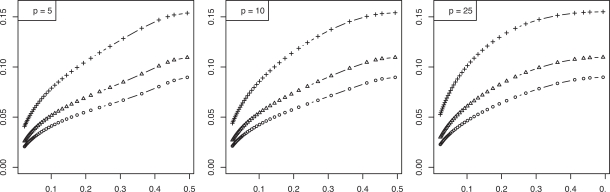
RMS *versus* Bayes error for leave-one-out in a Gaussian model: (+) is *n* = 20; (Δ) is *n* = 40; (o) is *n* = 60.

**Fig. (4):**
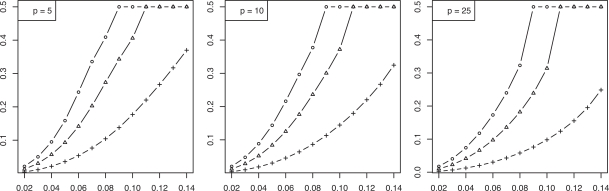
Maximum Bayes error *versus* RMS = λ for leave-one-out in a Gaussian model: (+) is *n* = 20; (Δ) is *n* = 40; (o) is *n* = 60.

**Fig. (5):**
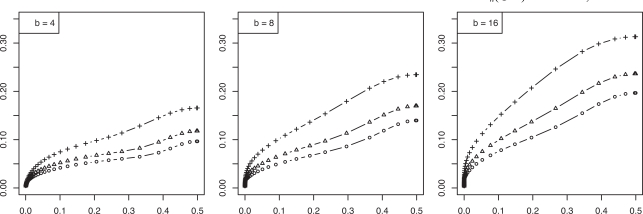
RMS *versus* Bayes error for resubstitution for discrete classification: (+) is *n* = 20; (Δ) is *n* = 40; (o) is *n* = 60.

**Fig. (6):**
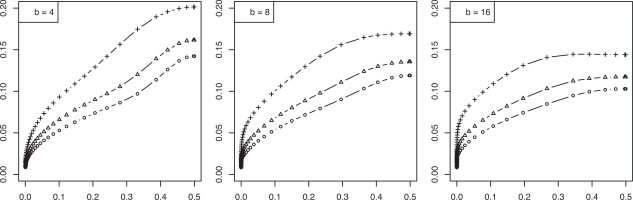
RMS *versus* Bayes error for leave-one-out for discrete classification: (+) is *n* = 20; (Δ) is *n* = 40; (o) is *n* = 60.

**Fig. (7):**
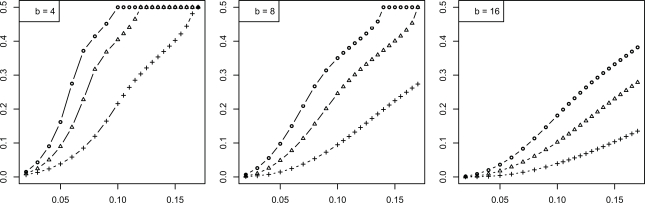
Maximum Bayes error *versus* RMS = λ for resubstitution for discrete classification: (+) is *n* = 20; (Δ) is *n* = 40; (o) is *n* = 60.

**Fig. (8):**
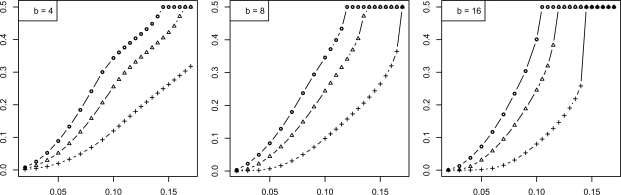
Maximum Bayes error *versus* RMS = λ for leave-one-out for discrete classification: (+) is *n* = 20; (Δ) is *n* = 40; (o) is *n* = 60.

**Fig. (9):**
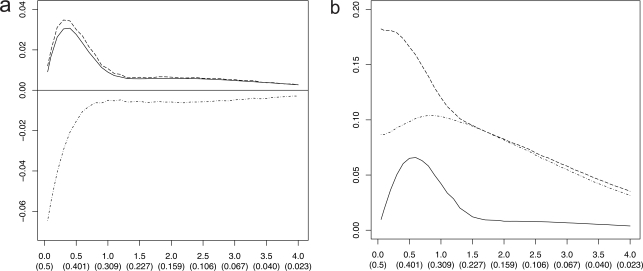
Error estimator performance for LDA in one-dimensional Gaussian model based on Glick’s paper; (**a**) *E*[ε_*LDA*_(*m*)] - ε_*bay*_(*m*) (solid), $E\left[{\overset{\wedge }{\varepsilon }}^{\mathrm{res}}\left(m\right)\right]-\varepsilon_{\mathrm{bay}}\left(m\right)$ (dots), and $E\left[{\overset{\wedge }{\varepsilon }}^{\mathrm{loo}}\left(m\right)\right]-\varepsilon_{\mathrm{bay}}\left(m\right)$ (dashes); (**b**) standard deviations for ε_*LDA*_(*m*) (solid), ${\hat{\varepsilon }}^{\mathrm{loo}}(m)$ (dots), and ${\hat{\varepsilon }}^{\mathrm{res}}(m)$ (dashes).

**Fig. (10):**
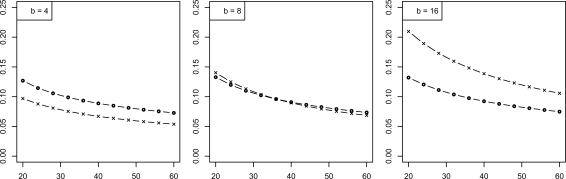
RMS *versus* sample size for discrete classification: (x) resubstitution; (o) leave-one-out.
